# Role of Neodymium Double Frequency Laser Posterior Hyaloidotomy in Delayed Presentation of Sub-hyaloid Haemorrhage

**DOI:** 10.7759/cureus.21534

**Published:** 2022-01-23

**Authors:** Alka Tripathi, Richa Agarwal, Sushma Chaurasia

**Affiliations:** 1 Ophthalmology, All India Institute of Medical Sciences, Gorakhpur, Gorakhpur, IND; 2 Ophthalmology, All India Institute of Medical Sciences, Gorakhpur, IND

**Keywords:** sub-hyaloid haemorrhage, outpatient procedure, valsalva retinopathy, nd yag laser, laser hyaloidotomy

## Abstract

Laser hyaloidotomy with Nd: YAG or green argon laser is a non-invasive, out-patient procedure in which the posterior hyaloid face is punctured and the opening enables the drainage of sub-hyaloid haemorrhage into the vitreous. The blood is absorbed gradually and vision is improved within days with gradual clearance of the macular area. Researchers recommend laser treatment in recent haemorrhages (<2 weeks) while for delayed cases invasive surgeries are advised. We report a case of delayed presentation of sub-hyaloid haemorrhage (>3 weeks) treated successfully with laser hyaloidotomy.

## Introduction

Sub-hyaloid haemorrhage or preretinal haemorrhage is a vitreous haemorrhage present in the sub-hyaloid space. Sub-hyaloid is a potential space between the hyaloid face and the internal limiting membrane and blood collected in this space resembles hyphema [1]. It is often boat-shaped involving the macula or adjacent areas causing profound loss of vision. The causes of sub-hyaloid haemorrhage are vascular disorders of the retina such as proliferative diabetic retinopathy, retinal vein occlusion, macro aneurysm, age-related macular degeneration, arterio-venous communication of the retina, myopia, trauma, haematological disorders such as aplastic anaemia and leukaemia, Valsalva retinopathy, Terson’s syndrome and Purtscher’s retinopathy [2-4]. Spontaneous resolution can occur but the process takes several months and it may cause degenerative changes of macula, proliferative vitreoretinopathy, and epiretinal membrane formation leading to irreversible loss of vision [1]. Therefore, early intervention in form of laser hyaloidotomy/membranotomy (Q-switched or 532 nm green laser), intravitreal injection of gas, and tissue plasminogen activator in case of submacular haemorrhage in ARMD, and pars plana vitrectomy for long-standing or complicated cases are advised [3,4].

Laser hyaloidotomy with Nd: YAG or green argon laser is a non-invasive method in which the posterior hyaloid face is punctured and the opening enables the drainage of sub-hyaloid haemorrhage into the vitreous. The blood is absorbed gradually and vision is improved within days with gradual clearance of the macular area [5,6]. Kroll and Busse recommended laser treatment within three to five days after the haemorrhage while for delayed cases invasive surgeries are advised [7]. We report a case of delayed presentation of sub-hyaloid haemorrhage (>3 weeks) treated successfully with laser hyaloidotomy.

## Case presentation

**Figure 1:**
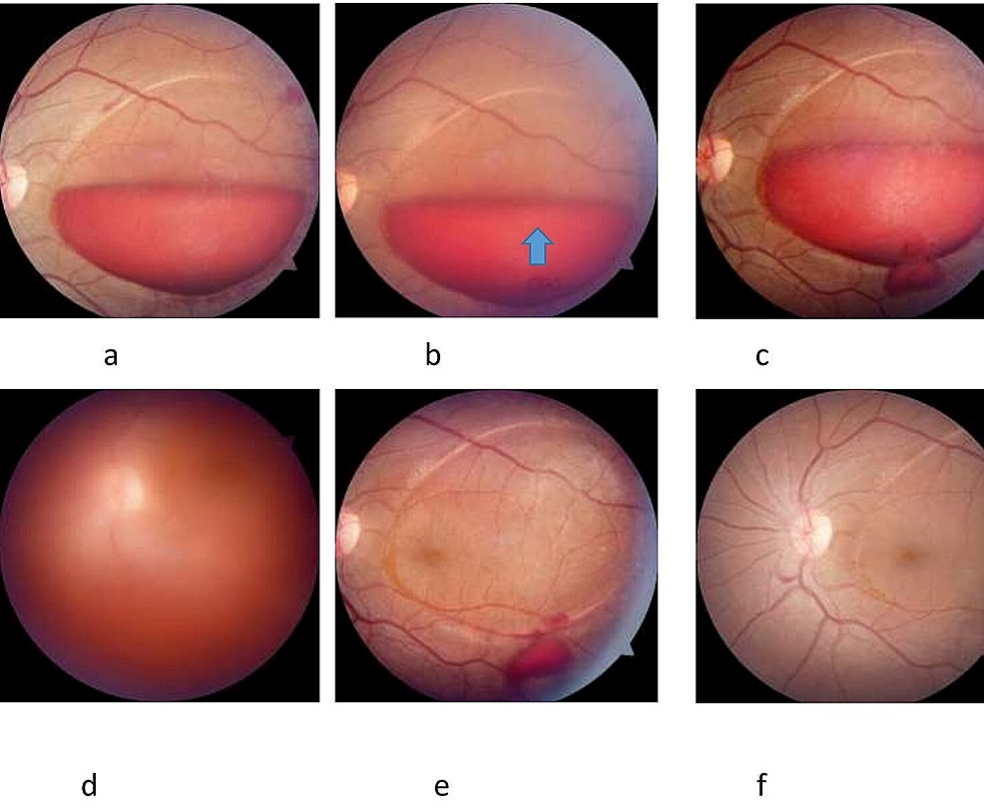
Fundus photographs. (a) >2 Disc diameter sub-hyaloid haemorrhage involving macula, (b) three laser spots visible with no bleed (arrow), (c) at fifth day follow up, (d) vitreous haze after second sitting, (e) at 15th day follow up, and (f) at one month follow up.

A 19-year-old female presented to the outpatient department of ophthalmology with sudden painless loss of vision in the left eye for 25 days. There was a history of repeated episodes of vomiting followed by a decrease in vision. Routine investigations including complete blood count (CBC), coagulation profile, and peripheral blood smear were within normal limits. She gave a history of similar episodes of repeated vomiting while traveling on multiple occasions. Visual acuity (VA) was OD 20/20 and OS hand movement (HM). Slit-lamp findings were unremarkable. Fundus examination revealed a large sub-hyaloid haemorrhage (>2 disc diameter) involving macula in the left eye (Figure1a) Right eye fundus was normal. She was advised laser hyaloidotomy with possibilities of pars plana vitrectomy in case of failure.

Procedure

All laser treatment were performed under mydriasis with tropicacyl plus (Phenylephrine (5% w/v) + Tropicamide (0.8% w/v) )eye drop and topical anaesthesia with 0.5% proparacaine (Paracaine) eye drop. Goldman 3-mirror contact lens (Volk) was used. Q-Switched Nd: YAG laser (Ellex Ultra Q) at energy level between 5 and 8 mJ with a single burst was used, and three openings were made on the anterior hyaloid surface along the inferior margin of the haemorrhage. There was no leakage of blood but the laser marks were visible (Figure 1b). The patient was called after five days and minimum leakage was seen on fundus examination (Figure 1c). The VA improved to 20/200. The second sitting of the laser was advised and two laser shots at 6 mJ power were given. Figure 1d shows the vitreous haze after laser. At one week follow up the VA in the left eye was 20/20 and the patient is doing fine at one month follow up (Figures 1e, 1f).

Post-procedure (after both settings) the patient was given topical non-steroidal anti-inflammatory agents four times a day for seven days.

## Discussion

Valsalva retinopathy was first described by Thomas Duane in 1972 [8]. Duane classified retinopathy into forwarding retinopathy (involving arterial circulation), backward retinopathy (involving venous circulation), and mixed retinopathy (involving arterial and venous circulation) [8]. Valsalva retinopathy is backward retinopathy. The venous system rostral to the heart lacks valves. Any sudden increase in the central venous pressure due to Valsalva manoeuvre (raised intrathoracic or intra-abdominal pressure against a closed glottis) is transmitted right up to the retinal capillaries which may rupture leading to preretinal/sub-hyaloid haemorrhage at macula [8]. The haemorrhage can be large involving macula (sub-hyaloid) or multiple small haemorrhages can be seen on the posterior pole. The common causes of Valsalva stress are weight lifting, vomiting, coughing, sneezing, aerobic exercises, constipation, blowing musical instruments, straining, physical activities [9].

A large sub-hyaloid haemorrhage involving macula can cause profound visual loss, which might be a matter of anxiety, particularly in the younger age group. Moreover, in due course of time, retinal changes can cause irreversible loss of vision. Therefore, early intervention is advised by most researchers. Laser hyaloidotomy or membranotomy with Nd: YAG or green argon laser is a non-invasive, cheap, daycare procedure with minimum side effects in expert hands. The blood leaks into the vitreous and is absorbed gradually and vision is improved within days. Faulborn in 1988 described Nd: YAG membranotomy as a procedure to drain premacular subhyaloid haemorrhage [10]. Thereafter many studies have been reported in the literature stating the usefulness of Nd: YAG membranotomy and energy levels used ranged from 2.5 to 50 mJ, with good results. Gabel et al stated that energy levels up to 50 mJ caused no retinal injury [11]. This proves that pre-retinal blood acts as a cushion and protects the underlying retina from any damage. Ulbig et al. recommended Nd: YAG laser treatment for recent premacular subhyaloid haemorrhage, which are >3 disc diameters in the area so that the fovea is protected from the photo disruptive effect of the laser and the energy level should not exceed beyond 9 mJ [11]. It is advisable to slowly increase the energy levels and the treatment location should be closest to the inferior edge of the haemorrhage.

The timing for Nd: YAG membranotomy is crucial as pre-retinal haemorrhage may clot in long-standing cases leading to failure of the procedure [12]. In a study by Khadka et al. two patients with symptoms of more than 45 days were treated successfully with Nd: YAG laser hyaloidotomy and concluded that the state of blood in the sub-hyaloid area is more important rather than the duration of haemorrhage [13]. However, Ulbig et al failed to drain clotted premacular sub-hyaloid haemorrhage of 35 days duration and thus advocate Nd: YAG membranotomy in recent cases only (<2 weeks) [11]. Better results with minimal complications have been seen in cases of sub-hyaloid haemorrhage due to Valsalva retinopathy as compared to other aetiologies. This might be due to the reason that patients of Valsalva retinopathy are young, healthy, and have no other co-morbidities [13].

Nd: YAG membranotomy is a safe procedure but peripheral retinal breaks, macular hole, and retinal detachment have been reported in some cases [14]. Ulbig et al. reported a macular hole in a young woman with Valsalva retinopathy after Nd: YAG laser treatment. The reason quoted was that photo disruptive effect was due to the laser being too close to the macula. In the same study retinal detachment was reported in 1 case with bilateral myopia and peripheral retinal breaks [11]. After hyaloidotomy epiretinal membrane formation and internal limiting membrane, the contraction has been reported in some cases by Kuruvilla et al. and Ahmadabadi et al. [12,15]. However, most of the researchers found no complications even after six months of the procedure.

Some universal recommendations for Nd: YAG membranotomy are A) the site chosen for membranotomy should be distant from the fovea and blood vessels preferably on the inferior most part of haemorrhage. B) One should start from low energy levels (5 mJ) and then gradually increase by 1-2 mJ up to a maximum of 12 mJ. If the blood does not drain out the patient can be called for the next sitting and on the failure of same, the procedure can be abandoned and PPV advised. (C) This procedure should be tried for haemorrhage >3 DD in size.

## Conclusions

Laser hyaloidotomy or membranotomy with Q-switched or 532 nm green laser is a safe, non-invasive, out-patient method advised for early presentation of sub-hyaloid haemorrhage (<1 week). However, cases with delayed presentation (>3 weeks) can also undergo this procedure with caution thus avoiding invasive surgeries. Good clinical assessment, precise positioning of the laser spots, and using lower energy levels are the key to a successful and safe procedure.
